# Supplementary material for: Gene-by-Environment Interactions Involving Maternal Exposures with Orofacial Cleft Risk in Filipinos
Source: Genes (Basel). 2025 Jul 25;16(8):876. doi: 10.3390/genes16080876 (PMC12386167; doi:10.3390/genes16080876)
Supplement: Supplementary file 1 [file genes-16-00876-s001.zip › Supplemental_Figures_Genes.pdf]

Supplemental Information

Supplemental Information includes four figures.

Figure S1 Sample flowcharts for the discovery sample (A) and the replication samples (B).

Figure S2 The first five principal components of ancestry (PCA) within the discovery cohort.

Figure S3 Manhattan plots (left) and quantile-quantile (QQ) plots (right) for genome-wide GEI scan with maternal smoking in the discovery cohort.

Figure S4 Manhattan plots (left) and quantile-quantile (QQ) plots (right) for genome-wide GEI scan with maternal vitamin use in the discovery cohort.

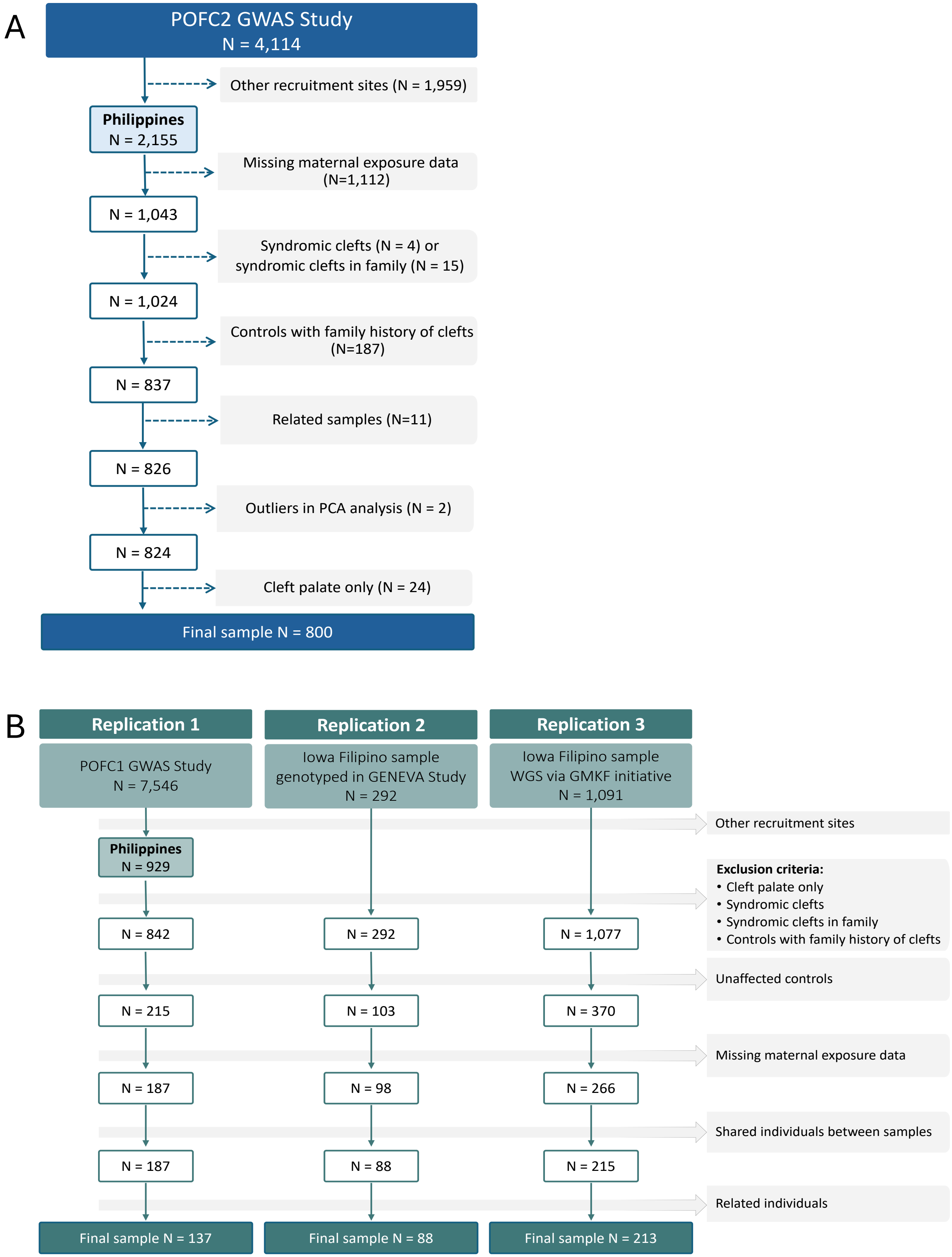

Figure S1 Sample flowcharts for the discovery sample (A) and the replication samples (B)

This study focused on individuals recruited in Philippines. The exclusion factors and the count of removed individuals are indicated in the light grey boxes.

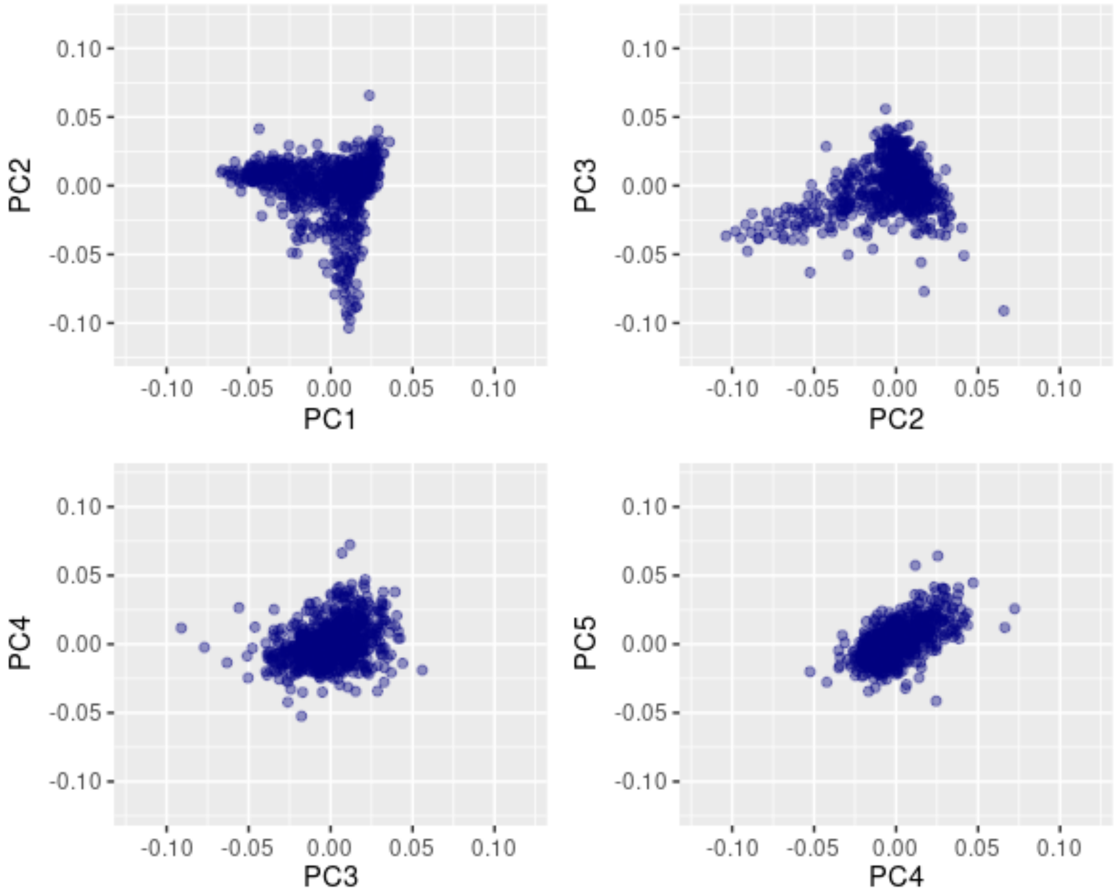

Figure S2 The first five principal components of ancestry (PCA) within the discovery cohort.

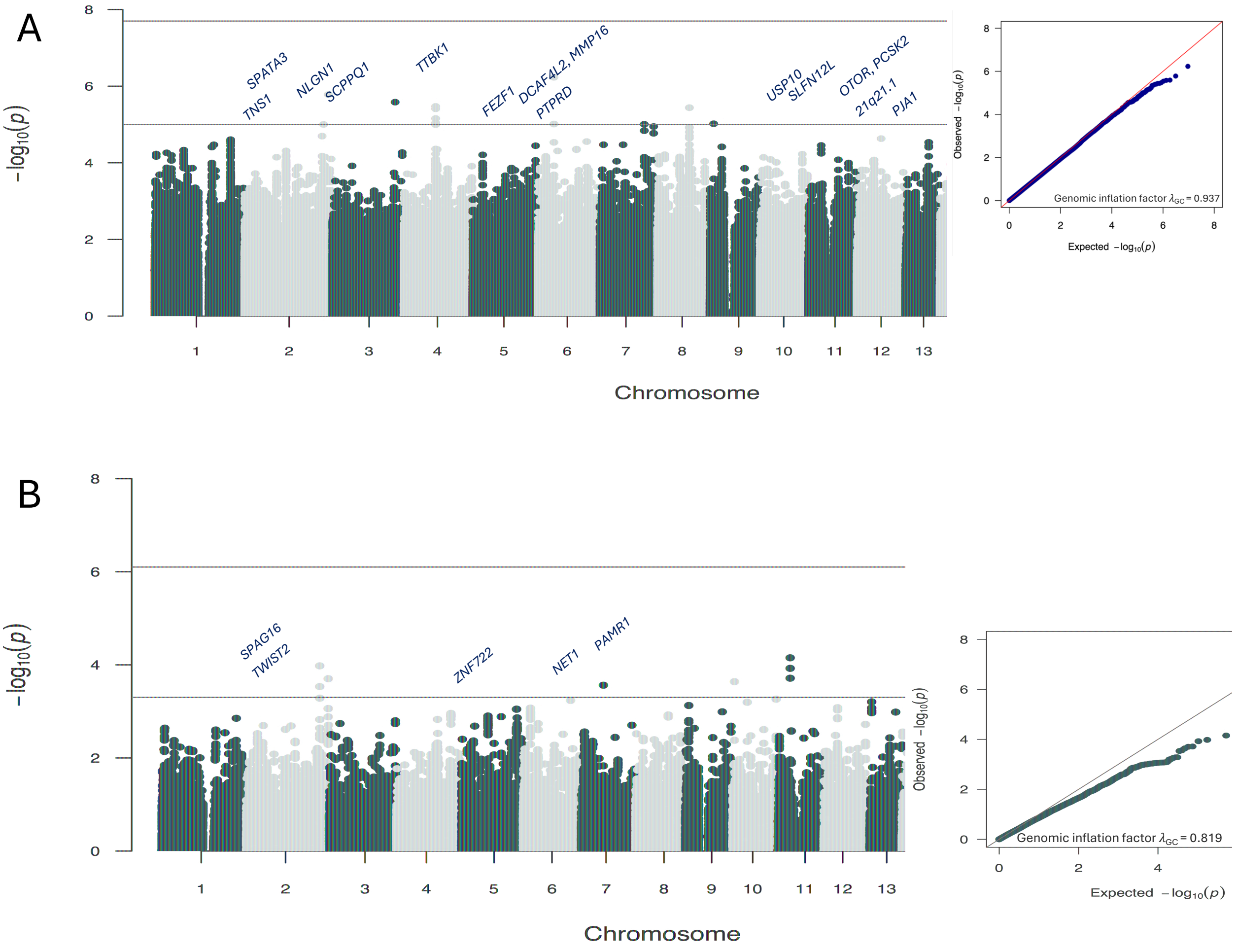

Figure S3 Manhattan plots (left) and quantile-quantile (QQ) plots (right) for genome-wide GEI scan with maternal smoking in the discovery cohort.

(A) 3df joint test with smoking and (**B**) two-step EDGE method results depicting the -log$_{10}$(p_{GxE}) from the step 2. The red lines indicate the Bonferroni-corrected genome-wide significance thresholds for 3df test (p_{3df}= 5 × 10^{-8}/3 ≈ 2 × 10^{-8}) and two-step EDGE method (p_{GxE}≈ 8×10^{-7}), respectively. The blue lines are the suggestive thresholds p_{3df}= 1 × 10^{-5} and p_{GxE} = 5 × 10^{-4}, respectively. The lead variants are annotated with the nearby gene(s) and are mapped to the chromosomal location on the x-axis.

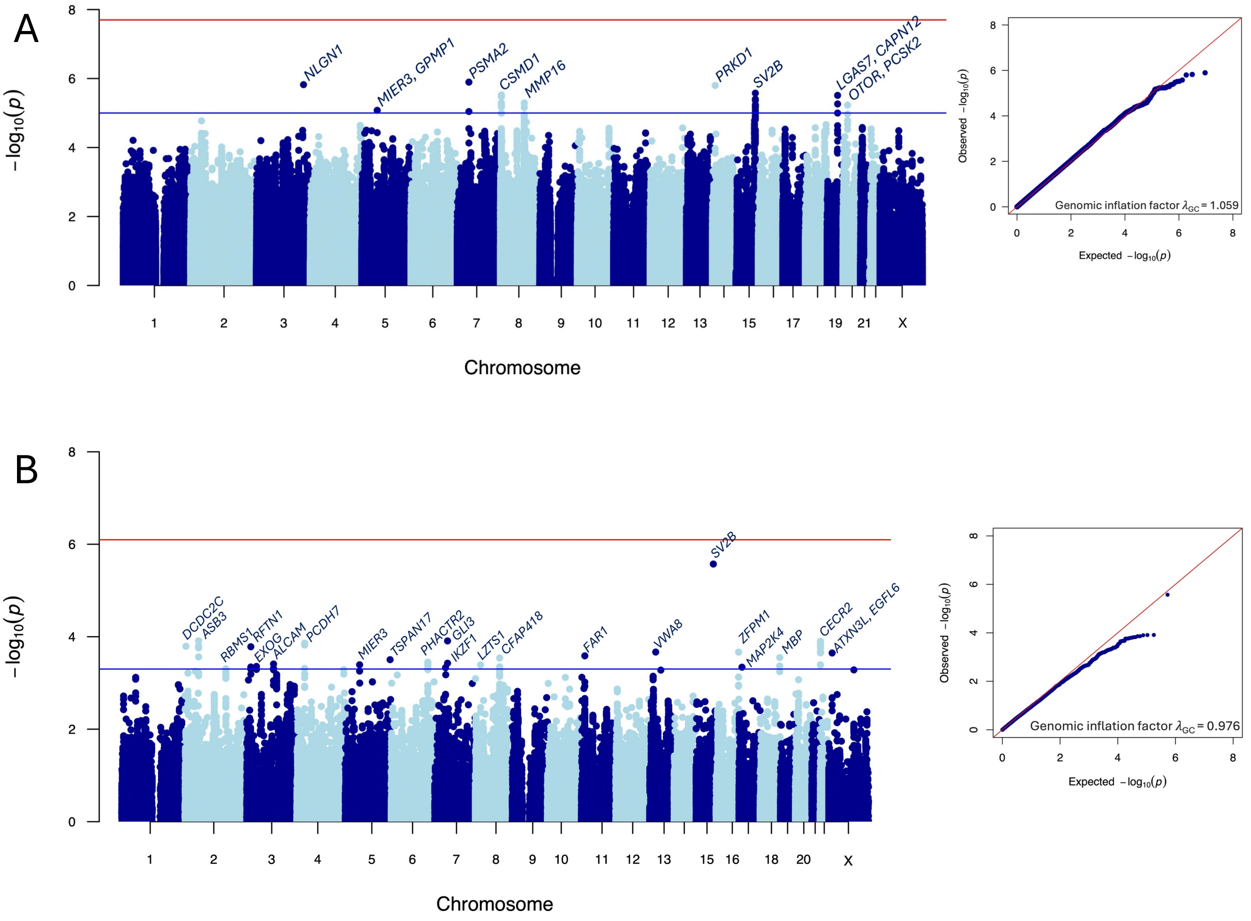

Figure S4 Manhattan plots (left) and quantile-quantile (QQ) plots (right) for genome-wide GEI scan with maternal vitamin use in the discovery cohort.

(A) 3df joint test with vitamin use and (**B**) two-step EDGE method results depicting the -$\log_{10}(p_{GxE})$ from the step 2. The red lines indicate the Bonferroni-corrected genome-wide significance thresholds for 3df test ($p_{3df} = 5 \times 10^{-8}/3 \approx 2 \times 10^{-8}$) and two-step EDGE method ($p_{GxE} \approx 8 \times 10^{-7}$), respectively. The blue lines are the suggestive thresholds $p_{3df} = 1 \times 10^{-5}$ and $p_{GxE} = 5 \times 10^{-4}$, respectively. The lead variants are annotated with the nearby gene(s) and are mapped to the chromosomal location on the x-axis.
